# Detection of Surface and Subsurface Flaws with Miniature GMR-Based Gradiometer

**DOI:** 10.3390/s22083097

**Published:** 2022-04-18

**Authors:** Huu-Thang Nguyen, Jen-Tzong Jeng, Van-Dong Doan, Chinh-Hieu Dinh, Xuan Thang Trinh, Duy-Vinh Dao

**Affiliations:** 1 Department of Mechanical Engineering, National Kaohsiung University of Science and Technology, Kaohsiung 807618, Taiwan; i108142110@nkust.edu.tw (H.-T.N.); i108142108@nkust.edu.tw (V.-D.D.); i108142107@nkust.edu.tw (C.-H.D.); i108142113@nkust.edu.tw (D.-V.D.); 2 Faculty of Mechanical Engineering, Hung Yen University of Technology and Education, Hung Yen 160000, Vietnam; xttrinh@utehy.edu.vn

**Keywords:** nondestructive testing, eddy current, giant magnetoresistance, defect detection

## Abstract

The eddy-current (EC) testing method is frequently utilized in the nondestructive inspection of conductive materials. To detect the minor and complex-shaped defects on the surface and in the underlying layers of a metallic sample, a miniature eddy-current probe with high sensitivity is preferred for enhancing the signal quality and spatial resolution of the obtained eddy-current images. In this work, we propose a novel design of a miniature eddy-current probe using a giant magnetoresistance (GMR) sensor fabricated on a silicon chip. The in-house-made GMR sensor comprises two cascaded spin-valve elements in parallel with an external variable resistor to form a Wheatstone bridge. The two elements on the chip are excited by the alternating magnetic field generated by a tiny coil aligned to the position that balances the background output of the bridge sensor. In this way, the two GMR elements behave effectively as an axial gradiometer with the bottom element sensitive to the surface and near-surface defects on a conductive specimen. The performance of the EC probe is verified by the numerical simulation and the corresponding experiments with machined defects on metallic samples. With this design, the geometric characteristics of the defects are clearly visualized with a spatial resolution of about 1 mm. The results demonstrate the feasibility and superiority of the proposed miniature GMR EC probe for characterizing the small and complex-shaped defects in multilayer conductive samples.

## 1. Introduction

In recent decades, the nondestructive testing (NDT) techniques based on the eddy-current (EC) effect have been widely applied in the manufacturing of conductive materials to ensure structural integrity and improve product quality. The main advantage of the eddy-current method is that it allows position-and-shape determination and the size estimation of defects on the conductive materials without any contact between the test specimen and the probe. It is especially important for the deep-lying defects that are not detectable by optical inspection. Various kinds of analytical, numerical, and experimental eddy-current techniques have been developed to detect and characterize the defects in a conductive sample, such as the calculation of the depth and opening width of a long crack [1], the analytic model of an ideal surface crack [2], and the impedance analysis of the coils for testing the surface crack based on the finite-element and boundary-element models [3,4]. The tiny coil-based probe can satisfy the requirement of high spatial resolution for detecting small defects [5,6]. However, the sensitivity is greatly reduced since the induced voltage depends proportionally on the magnetic flux through the pickup coil’s cross-section area [7]. Therefore, a sensor based on a small coil cannot meet the high-performance requirements and is only suitable for applications with high excitation frequencies. For both low- and high-frequency applications, the emerging method for evaluating structural integrity is an EC probe comprising magnetic field sensors, such as Hall sensors [8], superconducting quantum interference devices (SQUID) [9,10], giant-magnetoresistance (GMR) sensors, etc. Among them, a GMR sensor featuring high sensitivity, low cost, wide frequency range, and small size is a potential solution in many spheres, including nondestructive evaluation of metallic materials [11,12], cancer cell detection in biomedical testing [13], and electronic compasses in consumer electronics [14]. The most prominent applications of GMR sensors are the detection of tiny magnetic objects, as well as the imaging of defects in a conductive sample to characterize the shape, size, and depth of flaws and cracks. The rapid estimation of crack geometry and corrosion detection have been demonstrated by using the eddy-current probe with the bare-die [11,15,16,17] or packaged GMR sensor arrays [12]. The array probe enhances the inspection throughput, but the linear arrangement of the array makes it applicable only to a sample with a flat surface. In contrast, the EC probe with a single GMR sensor is more flexible. The reliability and capability of this kind of probe with various designs of excitation coils, including a ferrite core, long meander, planar, or flat spiral coils, have been demonstrated by many research groups [18,19,20,21,22]. The unsolved problem in such designs is the trade-off between spatial resolution and signal sensitivity, which cannot be resolved with the limited options in the coil-sensor arrangement set by the package size of the sensor and the diameter or thickness of the excitation coil. Besides, the performance of the big excitation coil in inspecting small defects is limited because of the low-efficiency interaction between the probe and the defect, which results in a decrease in the output amplitude [23]. In comparison with the absolute probes, the GMR-based EC probe in a gradiometer configuration exhibits higher sensitivity, as well as better spatial resolution. When the baselines are 0.3, 0.5, and 1 mm, the defect signals can be clearly resolved under the external field, ambient static field, and environmental disturbance [24,25]. The gradiometer EC probes comprising the other sensor technologies have also been developed and shown to exhibit high performance and good reliability, such as the SQUID gradiometer with an 8.5 mm baseline [26], the anisotropic magnetoresistance (AMR) gradiometers with baselines of 4 mm [27] and 40 mm [28], the magnetic tunnel junction (MTJ) gradiometer with a baseline of 40 mm [29], and the biaxial gradiometer with three GMR sensors vertically located with a baseline of 5 mm [30]. The axial gradiometer probe with a short baseline has a better immunity to the environmental field disturbance. For detecting deep-buried defects or metallic objects, a large baseline and a big excitation coil are preferred for enhancing the signal sensitivity, especially for the minor defects. However, the long baseline of an axial gradiometer results in a higher offset signal contributed by the defect-free sample surface. For the planar gradiometers [24,27], the increased baseline does not necessarily contribute to an increased offset level, but the crack-like defects cannot be detected with the planar gradiometer when the baseline is parallel to the orientation of the crack. In contrast, the defects of any planar orientation can be detected by the axial gradiometer probe.

In this work, a novel approach aiming at enhancing both the spatial resolution and the signal-to-noise ratio is suggested. A miniature EC probe based on the in-house-made GMR sensor in a half-bridge configuration and a tiny rectangular exciting coil is proposed. The chip is configured as an axial gradiometer with a baseline of 1.5 mm. The 1.8 mm opening of the tiny rectangular exciting coil fits well to the width of the GMR chip. With the closely packed chip-coil arrangement, the lift-off distance between the probe and the test sample is significantly minimized. The improvement in the spatial resolution and signal-to-noise ratio of the defect signals by using the proposed probe is analyzed and discussed. The proposed design is expected to improve the quality of the EC images in detecting the minor and complex-shaped defects on the surface and subsurface, such as machining tears, inclusions, and corrosion, of the metallic specimen or a short circuit in printed circuit boards.

## 2. Miniature Eddy-Current Probe with Spin-Valve GMR Sensor

### 2.1. Fabrication of GMR Chip in a Half-Bridge Configuration

**Figure 1:**
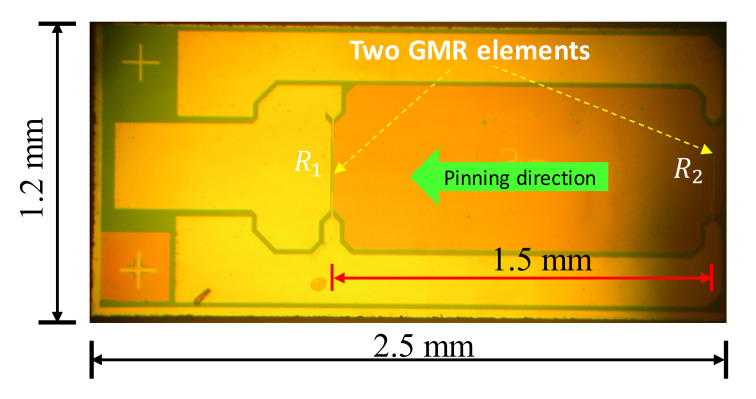
Top view and half-bridge layout of the fabricated GMR chip.

There are several implementations of a spin-valve GMR sensor fabricated on a single chip. With the unidirectional pinned field, the most feasible designs are the bridge layouts with two active and two inactive GMR elements. An example of the on-a-chip bridge design is the commercially available GMR sensor, GF708, from Sensitec GmbH, which comprises two inactive elements covered by magnetic shielding films and two active spin-valve elements forming a bridge configuration. In the present work, a novel half-bridge gradiometer with two cascaded active spin-valve elements on a chip is proposed. The fabrication method for the in-house-made GMR chip is similar to that reported in [14]. The spin-valve elements are based on the multi-layer structure of the metallic nanometer-thick films, including the ferromagnetic (FM) and nonmagnetic (MN) layers. The FM layers include the free (NiFe/CoFe) and pined (CoFe/IrMn) layers separated by the NM (copper) layer. The magnetization orientation of the pinned layer is stabilized by the IrMn film through the exchange bias. The magnetization orientation of the free layer is easily changed by the external magnetic field, thereby altering the current-in-plane resistance of the spin-valve element [31]. The distance between the reference element *R*_1_ and the sensing element *R*_2_ is 1.5 mm, which is the baseline length of the gradiometer, as shown in Figure 1. The spin-valve GMR elements are 0.4 mm in length and 3 μm in width. The pinning direction of the spin valve is parallel to the baseline and perpendicular to the length of the elements. The dimensions of the chip are 2.5 mm × 1.2 mm × 0.5 mm, respectively, for the length, width, and thickness. The zero-field resistance is *R*_2_ = 3.3 kΩ for the sensing element and *R*_1_ = 2.8 kΩ for the reference element. Although both the reference and sensing elements can detect the eddy-current signal, the output of the half-bridge is dominated by the change in *R*_2_ when the object under test is close to the eddy-current probe. The change in *R*_2_ is more significant because the minimum liftoff distance is much less than the baseline length of 1.5 mm.

### 2.2. Development of the EC Probe Based on a Miniature Axial Gradiometer

**Figure 2:**
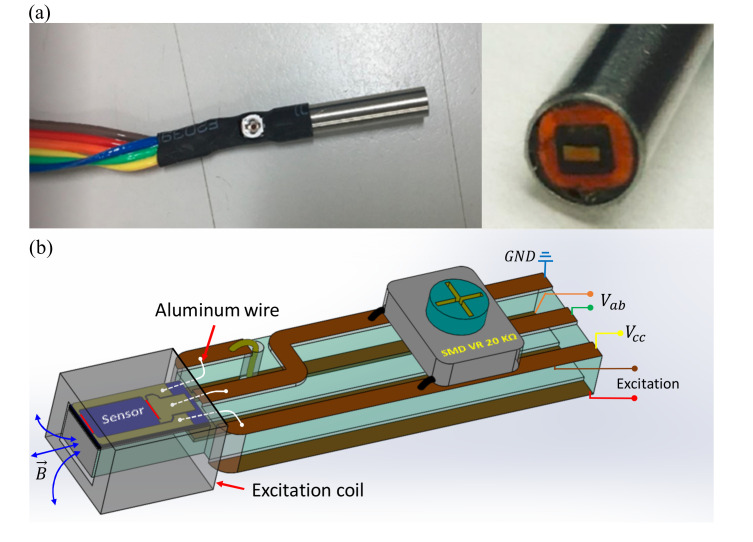
The gradiometer EC probe with a half-bridge GMR sensor chip. (**a**) Photographs of the encapsulated probe. The outer diameter of the stainless-steel housing tube is 4 mm. (**b**) The structure of the probe.

The miniature EC probe is constructed from the in-house-made half-bridge GMR chip as described above, a tiny printed circuit board (PCB), a 20 kΩ surface-mount-device (SMD) variable resistor, a small rectangular excitation coil, and a stainless-steel housing tube, as shown in Figure 2. The sizes of the excitation coil, such as the mean diameter, height, and thickness, are critical factors directly relating to the signal sensitivity and spatial resolution of the eddy-current probe. It has been reported that, for detecting small defects on the surface and underlying layers, the smaller coil is preferred [27,32] because the effective spatial range of the excitation field is smaller and hence the ratio between the defect size and the probe diameter increases, which results in an improvement in both sensitivity and spatial resolution. In the present study, the excitation coil is small in size and its specifications are given in Table 1. The outside dimensions are 2.9 mm × 3.1 mm and the height is 1.42 mm. The opening of the coil is 1.6 mm × 1.8 mm in dimension and fits well to the width of the half-bridge GMR chip, making it easy to adjust and locate the chip inside the excitation coil. A tiny double-sided PCB is made by an engraving machine to form the wirings and bonding pads for electrical connection with the GMR chip, as shown in Figure 2b. The half-bridge GMR chip is fixed to the tiny PCB so that the sensing direction is parallel to the length dimension of the PCB. The aluminum wire bonding method is used to electrically connect the GMR chip to the PCB. A 20 kΩ SMD variable resistor is connected in parallel to the GMR chip via the PCB to form a balanced bridge sensor, as shown in Figure 2. The background output signal of the probe can be minimized by tuning the variable resistor to avoid the saturation in the input voltage of the preamplifier. The excitation coil is fixed at the position where the sensing and reference elements receive a similar intensity of excitation. With this arrangement, the sensing direction of the GMR sensor is normal to the specimen surface. This means that only the vertical component (*B_z_*) of the secondary fields is detected by the probe. Although the primary magnetic field can also be detected, the contribution of the primary field is minimized by the balanced output of the gradiometer. The remaining unbalanced signal is the background output in the obtained eddy-current image. The constant background level resulting from the primary field has no effect on the image quality and hence can be subtracted from the data. Since the magnitude of *B_z_* is independent of the in-plane orientation of the magnetic source, the developed probe can detect the defect of any planar direction. The compact chip-and-coil arrangement simplifies the mechanical design of the EC probe.

**Figure 3:**
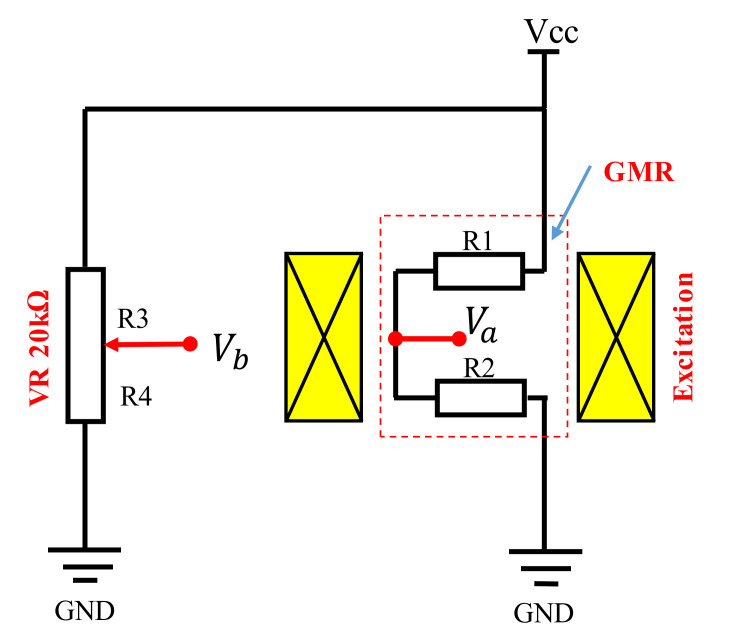
Circuit diagram of the developed probe consisting of the half-bridge GMR sensor chip and SMD VR 20 kΩ.

**Figure 4:**
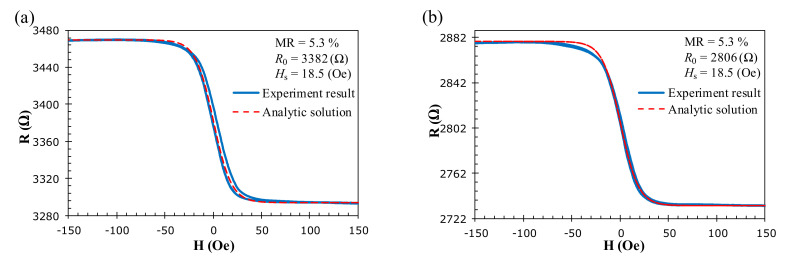
The MR curve for the spin-valve GMR elements: (**a**) the sensing element *R*_2_ and (**b**) the reference element *R*_1_.

The magnetic field measured by the native GMR elements is usually outside the linear working range because the offset field results from the exchange bias. To operate the sensor at the working point with the optimal sensitivity, the DC bias and AC excitation fields are generated by the same excitation coil at the same time, as shown in Figure 3. The DC current in the excitation coil shifts the working point to the center of the linear range where the sensitivity is maximized. The AC excitation amplitude is adjusted until the magnetic field induced is high enough to operate the probe with the best sensitivity to the flaw. The output voltage of the probe is the difference between the field-dependent half-bridge voltage *V_a_* and the static potential *V_b_* of the variable resistor:

(1) $$V_{ab}=V_{a}-V_{b}=V_{cc}\times \left(\frac{R_{2}(H)}{R_{1}(H)+R_{2}(H)}-\frac{R_{4}}{R_{3}+R_{4}}\right)$$

where *V_cc_* is the bias voltage, *H* is the applied magnetic field, *R*_2_ is the resistance of the sensing element, *R*_1_ is the resistance of the reference element, and *R*_3_ and *R*_4_ are the resistances of the variable resistor. The sensor output can also be predicted by relating the magnetoresistance with the applied magnetic field *H* as follows:

(2) $$R\left(H\right)=R_{0}\left(1+\frac{r}{r+2}\times \tanh\left(-\frac{H}{H_{s}}\right)\right),$$

where *R*_0_ is the resistance value at zero magnetic field, *H*_s_ is the saturation field, and *r* is the magnetoresistance (MR) ratio defined as:

(3) $$r=\frac{R_{P}-R_{AP}}{R_{P}}$$

where *R_P_* is the resistance of saturated parallel magnetization and *R_AP_* is the resistance of the antiparallel state. When |*H*| ≥ *H_s_*, the resistance of the GMR sensor will not immediately saturate to *R*_P_ or *R*_AP_. The MR ratio must be estimated for |*H*| ≥ 3*H_s_* according to the experimental data. The resistance-field (*R-H*) curve for the elements on the half-bridge GMR sensor was measured in a 1 Hz sweeping field of 150 Oe generated by an electromagnet with a current-to-field transfer coefficient of 1000 Oe/A. To set the operating point to the zero external field, a 37 Oe DC field is applied along with the AC excitation by the tiny rectangular excitation coil to shift the working point of the sensor. This makes the *R*-*H* curve of each GMR element become anti-symmetric about the zero magnetic field, as shown in Figure 4, where the red dashed lines are calculated by (2). It can be seen that the hyperbolic tangent function fits satisfactorily to the experiment data when the small hysteresis is neglected. The best-fit parameters are found to be *H_s_* = 18.5 Oe and *r* = 5.3% for both GMR elements. The resistance *R*_0_ at the zero magnetic field for the sensing and reference elements is 3.3 and 2.8 kΩ, respectively. 

## 3. Experiment Setup

### 3.1. Experimental Eddy-Current Flaw Detection System

**Figure 5:**
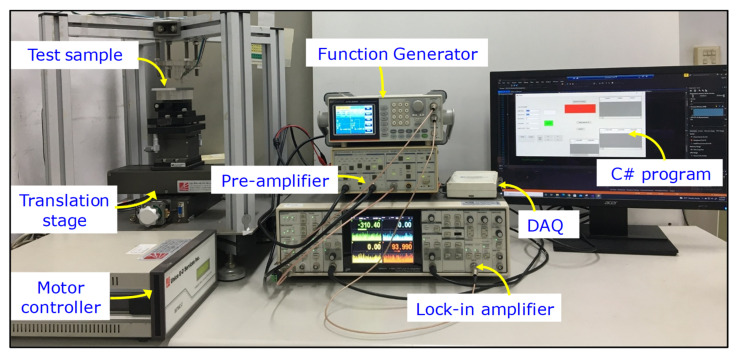
The automatic scanning system for the EC probe based on a half-bridge GMR sensor.

In the GMR eddy-current system, the excitation field is induced by injecting a sinewave signal into the excitation coil surrounding the GMR sensor. The amplitude of the excitation signal for the proposed probe can change from 1 V to 10 V in peak-to-peak values with various frequencies up to 1 MHz. In our experiments, the excitation coil is driven by a 6 V peak-to-peak AC voltage and a −1.3 V DC offset, while the excitation frequency is tuned to match the characteristics of the tested materials determined by the experiments. The typical root-mean-square (rms) amplitude for the excitation current is 57.6 mA at 40 kHz. The 1 V DC supply voltage (*V_cc_*) is generated by a circuit powered by a battery set. The lift-off distance between the probe and the specimen surface is 0.2 mm and the specimen moves under the probe with a step size of 0.125 mm. Figure 5 shows the main components of the experimental system including the output data processing system, data acquisition (DAQ) device, and the *x*-*y* positioning system. As the output voltage of the GMR sensor is on the order of a millivolt and vulnerable to wiring interference, the output signals of the probe are amplified and filtered by the low-noise pre-amplifier, model SR560 from Stanford Research System. The pre-amplifier output is analyzed by the lock-in amplifier, model SR865A from Stanford Research Systems. To read the output voltage of the sensor in real time while scanning the sample surface, a DAQ module USB-6216 from National Instruments was used to record the in-phase and quadrature eddy-current signals from the analog outputs of the lock-in amplifier. The sampling rate of the DAQ device is 400 kS/s. The scanning speed and sampling rate are adjusted by the C# program to provide an accurate representation of the eddy-current signals in response to the scanning. The probe is tightly fixed to avoid the interference induced by a bending of the signal cable in the scanning process, while the test sample is mounted on a motor-controlled two-axis translation stage, model 08TMC-2, from Unice E-O Services Inc. The sample is mounted on a height-and-tilt adjustment mechanism to minimize the lift-off variation. The stepping-motor controller is connected to a computer via a serial port. The operation parameters of the system, such as step size, scanning range, and velocity, as well as the data-recording process, are controlled by a self-developed C# program.

### 3.2. Kinds of Specimens under Test

**Figure 6:**
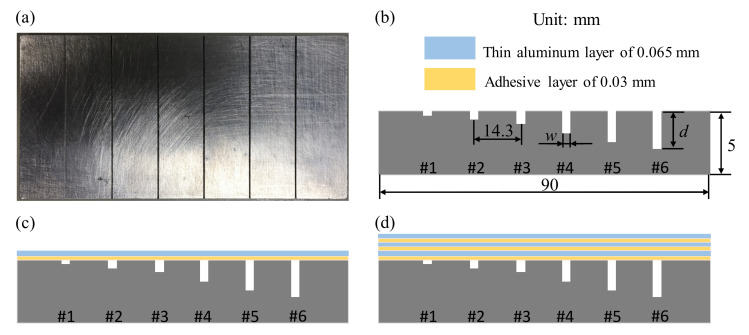
An aluminum plate with long machined cracks of different depths. (**a**) Photograph of aluminum sample. (**b**) Surface flaws. (**c**) Subsurface flaws with an aluminum tape layer attached. (**d**) Subsurface flaws with three aluminum tape layers attached.

To verify the performance of the designed probe for detecting surface and subsurface defects, the nondestructive inspection is implemented on three types of samples. The first kind of specimen is an aluminum plate 90 mm × 50 mm × 5 mm in dimension. The cracks on the specimen simulated by the machined slots of the same length (50 mm) and width (0.5 mm) are numbered as #1 to #6 for which the depths are respectively 0.1, 0.3, 0.5, 1.0, 1.5, and 1.8 mm, as shown in Figure 6b. The distance between the centers of adjacent cracks is 14.3 mm. For the inspection of surface defects, the probe is placed on the specimen surface with a lift-off distance of 0.2 mm. For subsurface flaws, the specimen surface is covered by one layer of aluminum tape, as shown in Figure 6c,d. Each layer of the aluminum tape consists of an adhesive layer of 0.03 mm and a thin aluminum layer of 0.065 mm. With one and three layers of aluminum tape, the buried depths of the machined cracks are respectively *h* = 0.095 and 0.285 mm, where *h* is defined as the total thickness of the tape layers on the sample surface.

**Figure 7:**
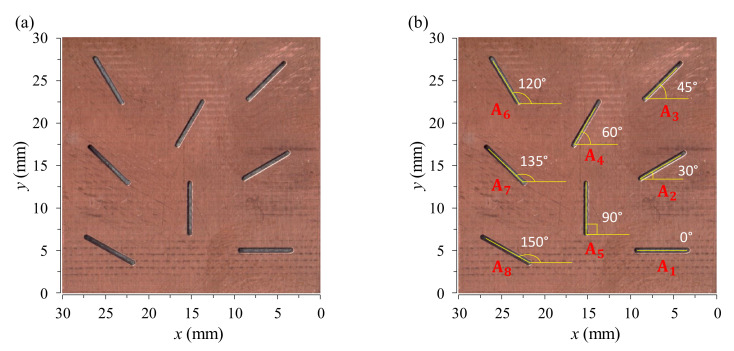
The PCB specimen for testing the performance of the EC probe in determining crack orientation: (**a**) photograph of the PCB with defects, and (**b**) illustration for the orientation angles of the defects with the defect numbers.

The second kind of specimen, which is used for exploring the performance in inspecting crack direction, is a copper film with machined slits on a 30 mm × 30 mm printed circuit board (PCB), as described in Figure 7. The eight slits simulating the cracks are made with the different angles of 0°, 30°, 45°, 60°, 90°, 120°, 135°, and 150° against the horizontal direction, while the length and width of the slits are constant at 6.5 mm and 0.6 mm, respectively.

**Figure 8:**
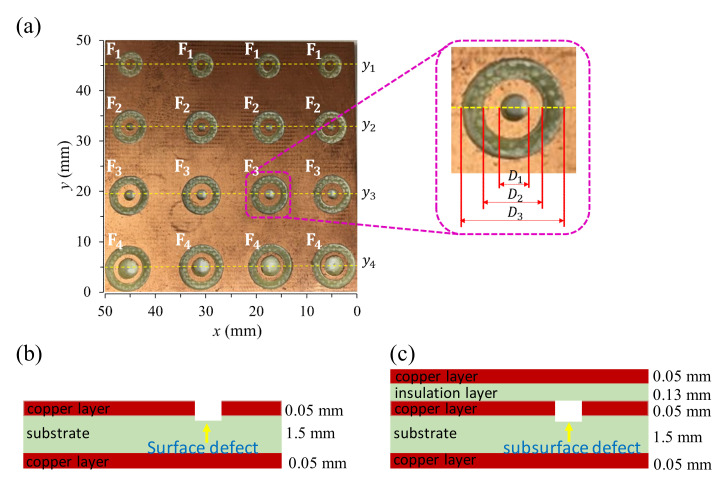
The multilayer PCB specimen with surface and subsurface defects: (**a**) photograph of the specimen and the geometrical dimensions of the machined flaws on the copper layer, (**b**) the machined flaws on the first layer of the two-layer PCB, and (**c**) the machined flaws on the second layer of the three-layer PCB.

The third specimen is a two-layer and three-layer square printed circuit board 50 mm × 50 mm in dimension with concentric circular and ring-shaped metal losses made by an engraving machine. The dimensions and arrangement of the machined flaws are depicted in Figure 8a and the geometrical dimensions of the flaws on the PCB are shown in Table 2. Each artificial flaw contains a central circular hole of diameter *D*_1_ and a ring-shaped flaw of inner and outer diameters *D*_2_ and *D*_3_, respectively, as described in Figure 8a. There are four kinds of flaws with different dimensions of *D*_1_, *D*_2_, and *D*_3_ that are designated as F_1_ to F_4_. Each kind of flaw has four in a row located on lines *y*_1_, *y*_2_, *y*_3_, and *y*_4_, respectively. The gradual increase in the dimensions of the machined flaws is designed to investigate the ultimate spatial resolution of the GMR-based flaw detector, of which the performance is highly correlated with its geometrical parameters. To evaluate the performance of the probe in detecting surface and subsurface defects, the machined flaws are, respectively, on the first and second copper layers of the two- and three-layer PCB samples, as shown in Figure 8b,c, for which the three-layer structure is made by attaching an insulation layer of 0.13 mm and a copper layer of 0.05 mm. The corresponding buried depth of the subsurface flaws is 0.18 mm below the top surface of the three-layer PCB.

## 4. Numerical Model and Signal Analysis

**Figure 9:**
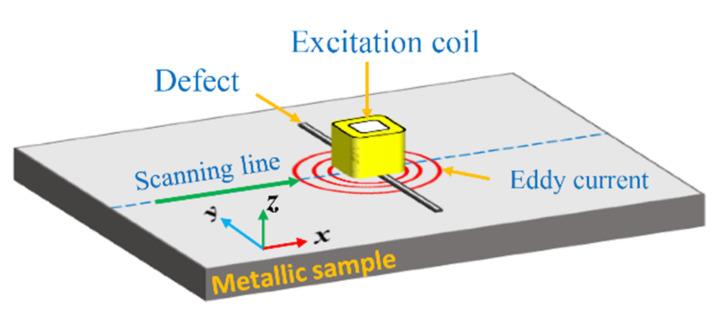
Simulation model for inspecting cracks with different depths on an aluminum plate. The excitation coil is placed above the sample surface with the lift-off distance of 0.2 mm.

To evaluate the performance, as well as the underlying operating principle, of the developed probe, the EC density on the test sample and the secondary magnetic field generated by the ECs are numerically analyzed using ANSYS MAXWELL software. The simulation model for the proposed probe is shown in Figure 9, where a rectangular excitation coil located above an aluminum sample with artificial cracks is constructed using the parameters in Table 1. The axis of the excitation coil is along the z-direction. The specimen containing defects is placed in the *x-y* plane with the lift-off distance of 0.2 mm. When the probe scans along the *x*-direction crossing the defect, the eddy current in the sample is induced by the excitation coil carrying a sinusoidal alternating current. These induced currents generate the secondary magnetic field, of which the component along the *z*-direction is detected by the GMR sensors. In the simulation, a 40 mA rms sinusoidal current of 40 kHz is injected into the excitation coil. The six cracks on the test specimen are 50 mm in length and 0.5 mm in width, while the depths are respectively 0.1, 0.3, 0.5, 1, 1.5, and 1.8 mm, as described in Figure 6a,b.

**Figure 10:**
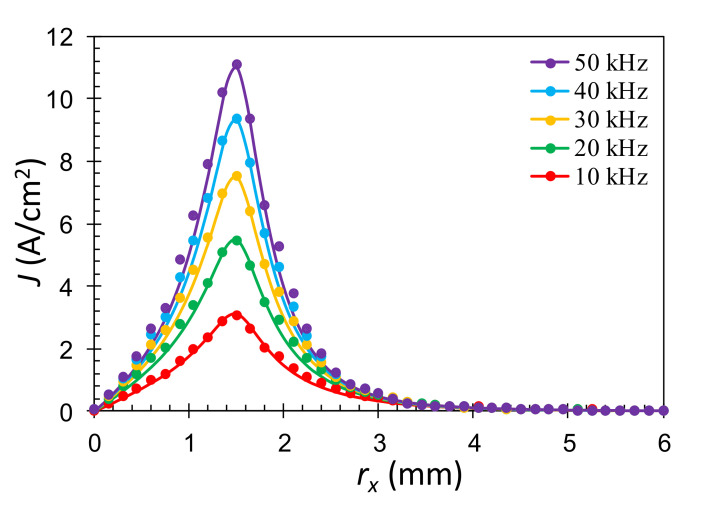
Amplitude of the eddy-current density along the x-axis on the surface of a flawless aluminum slab at frequencies of 10, 20, 30, and 40 kHz calculated by the MATLAB software (solid curves) and the ANSYS software (solid circles). The excitation coil has a radius of 1.5 mm, a height of 0.05 mm, and a lift-off distance of *L*_1_ = 0.2 mm. The surface current density of the excitation coil is ζ = 800 A/m.

To verify the validity of the finite-element analysis, a solenoid-coil model is employed to compare the induced eddy currents calculated by the finite-element software and the analytical solution. In this model, the solenoid excitation coil is above a flawless semi-infinite conductor with the conductivity of σ. The radial distribution of the EC density on the sample surface excited by the solenoidal-coil model can be derived from the solution of the filamentary circular excitation coil over a semi-infinite conducting sample by superposition [33]. The obtained analytic solution is:

(4) $$J(r_{x})=-j\frac{r_{e}\zeta }{\delta^{2}}\int_{0}^{\infty }J_{1}(\alpha r_{e})J_{1}(\alpha r_{x})\cdot \frac{2(e^{-\alpha L_{1}}-e^{-\alpha L_{2}})}{\alpha +\sqrt{\alpha^{2}+j(2/\delta^{2})}}d\alpha$$

where *r_x_* is the radial coordinate with the origin at the intersection of the coil’s axis and the sample surface, *r*_e_ is the coil radius, *L*_1_ = 0.2 mm is the lift-off distance between the coil bottom and the specimen surface, *L*_2_ = 0.25 mm is the distance from the coil top to the sample surface, *L*_2_ − *L*_1_ = 0.05 is the height of the coil, ζ (A/m) is the surface current density in the coil, *J*_1_(α*r*_e_) is the Bessel first-order function of the first kind, and δ is the skin depth defined as:

(5) $$\delta =\sqrt{\frac{1}{\pi f\mu \sigma }}$$

where *f* is the excitation frequency, μ is the permeability, and σ is the conductivity of the specimen. The prediction of Equation (4) can be calculated by performing numerical integration using the MATLAB software. The distribution of the simulated and calculated eddy-current densities on the aluminum specimen surface along the *x*-axis at the 10, 20, 30, and 40 kHz excitation frequencies is presented in Figure 10, where the solid curves are the eddy-current densities calculated by Equation (4) using the MATLAB software and the solid circle symbols are the results obtained by the ANSYS software for *r*_e_ = 1.5 mm. It can be observed that the amplitude of the eddy-current density is generally enhanced when the higher excitation frequencies are applied. Along the *r_x_* direction, the amplitude increases initially and reaches a peak near the coil radius, then the amplitude decreases to zero as *r_x_* goes to infinity. It can be seen that the results obtained from the analytic solution and the finite element method are well in agreement, indicating that the simulation based on the finite-element software is reliable in evaluating the EC distribution in conducting samples.

**Figure 11:**
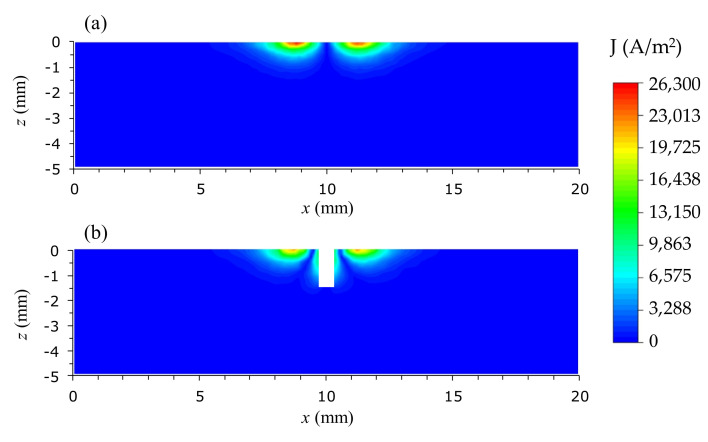
The eddy-current distribution following the depth of (**a**) a flawless aluminum sample and (**b**) a flawed sample at the excitation frequency of 40 kHz. The crack used in (**b**) has a 1.5 mm depth along the *y*-axis.

The simulated EC distribution in the unflawed aluminum sample and the flawed sample with a crack of 1.5 mm depth is shown in Figure 11. The EC density is concentrated at positions around the radius of the excitation coil and it drops rapidly for locations away from the radius of the excitation coil. It can be found that the EC density on the flawless aluminum slab in Figure 11a is higher than the EC density on the aluminum plate with the crack in Figure 11b because of disturbance caused by the crack defect. This leads to the variation of the secondary field induced by these ECs with the presence of the crack.

**Figure 12:**
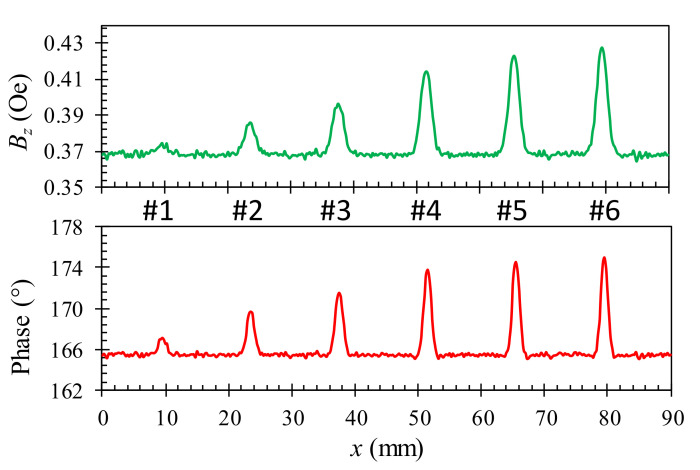
The amplitude and phase signals of the vertical component of secondary field when scanning over cracks with different depths of 0.1, 0.3, 0.5, 1.0, 1.5, and 1.8 mm with the 40 kHz excitation frequency. The lift-off distance is 0.2 mm.

For the novel probe design in the present work, the component of the secondary magnetic field orthogonal to the sample surface is detected by the GMR sensor; therefore, only this field component is analyzed in the simulation. To estimate the waveform of the output signals from the EC probe, the one-dimensional (1D) scanning process was simulated by moving the rectangular excitation coil through the surface slots over the aluminum specimen, which is similar to the sample depicted in Figure 6a,b, with a 0.25 mm step. The amplitude and phase angle of the EC signals detected by the sensing element are calculated and analyzed. Figure 12 shows the variation of the magnetic field component *B*_z_ for slots with different depths of 0.1, 0.3, 0.5, 1, 1.5, and 1.8 mm. It can be seen that the prominent peak values of the secondary magnetic field *B*_z_ occur near the cracks. The amplitude and phase angles of *B*_z_ are correlated with the depth of cracks. The simulated results show that the proposed probe is reliable to detect defects in the conducting material.

## 5. Experimental Result and Analysis

### 5.1. Lift-Off Effect of the Proposed Probe

**Figure 13:**
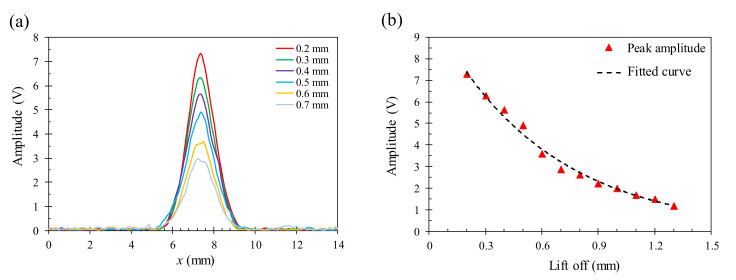
The signal change of the proposed probe on a 1.5 mm deep crack with different lift-off distances from 0.2 to 1.3 mm at the 45 kHz excitation frequency: (**a**) amplitude distribution near the crack, (**b**) reduction in the peak amplitude with increasing lift-off.

The lift-off distance is an important factor that strongly affects the quality of the EC images obtained by the GMR-based probes in nondestructive testing. For the GMR probes with a small excitation coil, the sensitivity to the defects in conductive materials significantly depends on the change in the lift-off distance between the probe and the sample. To explore the lift-off effect, the response of the proposed probe for a 1.5 mm deep crack on the aluminum specimen is collected at different lift-off distances from 0.2 to 1.3 mm with a 0.1 mm step size. The result in Figure 13 shows that the obtained amplitude is significantly reduced by 84% when the liftoff distance increases from 0.2 to 1.3 mm. Besides, the peak of the signals is also distorted and the obtained signals are noisier with the increasing lift-off distance. For the samples with an uneven surface, a proper algorithm to make a correction to the lift-off effect would be necessary. To avoid the possible error arising from the lift-off effect in the subsequent experiments, the lift-off distance is set at 0.2 mm so as to avoid the contact between the probe bottom and the sample surface. In addition, the inclination of the scanning surface with respect to the specimen surface is minimized by carefully adjusting the level of the sample.

### 5.2. Frequency Effect for Surface and Subsurface Defect Detection

The design goal of the EC probe in the present work is to identify the characteristics of the defect such as the geometrical dimensions and position, as well as orientation, depth, etc. To achieve the best performance of the proposed probe for detecting defects in specific cases, it is essential to operate the probe at the optimal excitation frequency to maximize the signal response so that the defect information can be clearly observed to reconstruct the geometry. The optimal frequency is determined by the experiments in which the in-phase (Re) and quadrature (Im) output signals are recorded for the cracks buried at different depths over a range of frequencies to calculate the corresponding amplitude and phase values. For the multilayer PCB samples, cracks 0.8 mm in width and 15 mm in length are located respectively on the first and second layers of the two- and three-layer PCBs. The excitation frequency for flaw detection varies from 15 kHz to 125 kHz with an increment of 5 kHz. For the aluminum sample, the crack of 0.5 mm width and 1.5 mm depth buried at *h* = 0, 0.095, and 0.285 mm is inspected in the frequency range from 5 kHz to 50 kHz in steps of 5 kHz.

**Figure 14:**
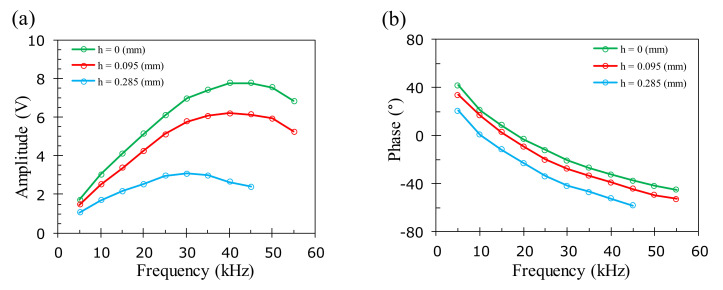
The amplitude and phase response of the EC signals on a crack with a 0.5 mm width and 1.5 mm depth buried at *h* = 0, 0.095, and 0.285 mm on the aluminum plate with increasing frequencies. (**a**) Peak amplitude and (**b**) corresponding phase.

The optimal operation frequency is affected by many factors, including the skin depth, conductivity, and permeability of the material, as well as the shape and buried depth of the defects. Figure 14 shows the amplitude and phase of the EC signal at the location of the surface and buried cracks in the aluminum samples at different excitation frequencies. It is observed that when increasing the excitation frequency, the amplitude increases initially but gradually reduces beyond the optimal frequency, as shown in Figure 14a. The phase reduces continuously with increasing frequency, as presented in Figure 14b. The results show that the optimal excitation frequency of the aluminum samples for the cracks buried at *h* = 0, 0.095, 0.285 mm is 45, 40, and 30 kHz. The corresponding skin depths are 0.38, 0.41, and 0.47 mm, respectively, for the optimal frequencies of 45, 40, and 30 kHz. The existence of the optimal excitation frequency and its reduction with the buried depth can be explained qualitatively by the skin effect [34]. The secondary magnetic field at the sensor’s position is contributed to by all of the ECs in the test sample following the superposition principle. Therefore, the magnitude of the secondary magnetic field increases as long as the ECs are enhanced at the sample surface with an increasing excitation frequency. At the higher frequencies, the ECs decay strongly with an increasing depth and hence lead to a reduction in the secondary magnetic field induced by ECs around the buried flaws.

**Figure 15:**
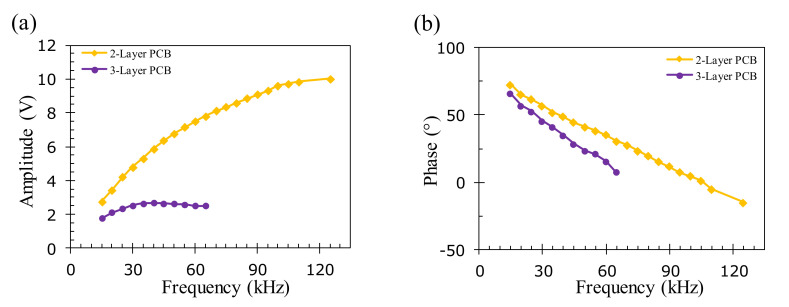
The amplitude and phase response of the EC signals on a crack with a 0.8 mm width and 15 mm length located respectively on the first and second layers of the two- and three-layer PCB samples with increasing frequencies. (**a**) Peak amplitude and (**b**) corresponding phase.

The effect of the excitation frequency on the EC signal for the two- and three-layer PCB samples is shown in Figure 15, where the optimal excitation frequency is above 125 kHz for the surface crack on the two-layer PCB, which is higher than the optimal frequency of the aluminum sample. The higher optimal frequency can be attributed to the much smaller thickness of the copper layer, which is only 0.05 mm. Namely, the geometric center of the crack is only 0.025 mm below the sample surface, which is closer to the bottom of the EC probe in comparison with the geometric center of the simulated flaw on the aluminum sample. The phase decreases linearly when increasing the excitation frequency for the surface and subsurface flaws on the two- and three-layer PCB samples, as shown in Figure 15b. In the subsequent experiments for inspecting the surface defects on the two-layer PCB samples, the excitation frequency is chosen to be 60 kHz to maximize the signal-to-noise ratio to ensure that the defect information can be still clearly extracted. The corresponding skin depth is 0.27 mm, which is larger than the 0.1 mm total thickness of the copper layers on the two-layer PCB. For testing the crack buried at the depth of 0.18 mm on the three-layer PCB, the optimal excitation frequency is found to be 40 kHz. The corresponding skin depth at this frequency is 0.33 mm, which is larger than the 0.15 mm total thickness of copper layers on the three-layer PCB sample.

### 5.3. Detection Limit: Surface and Subsurface Flaws on Aluminum Specimen

**Figure 16:**
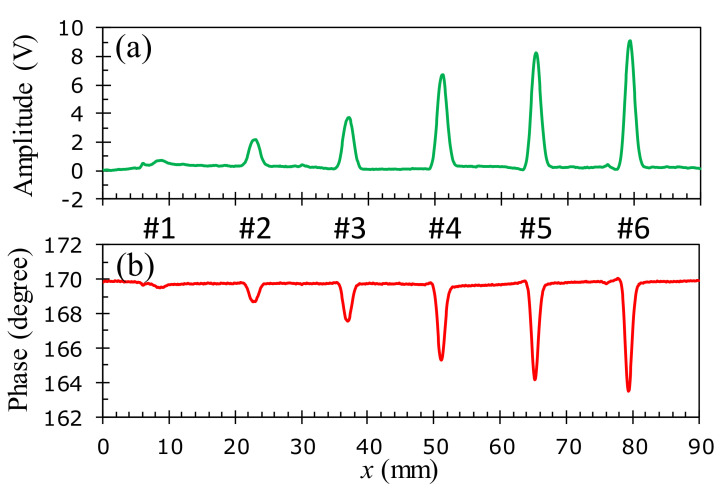
Inspection of surface cracks (*h* = 0 mm) at the 45 kHz optimal excitation frequency on an aluminum specimen: (**a**) amplitude variation, (**b**) phase angle variation.

**Figure 17:**
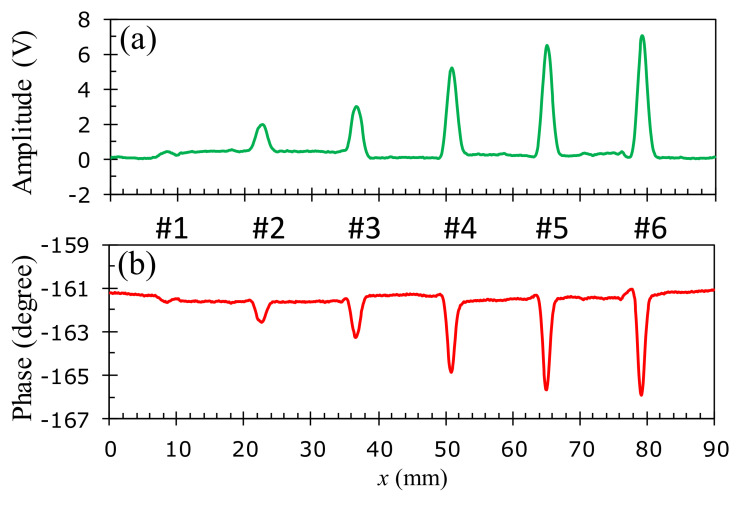
Inspection of cracks buried at *h* = 0.095 mm at the 40 kHz optimal excitation frequency on an aluminum specimen: (**a**) amplitude variation, (**b**) phase angle variation.

**Figure 18:**
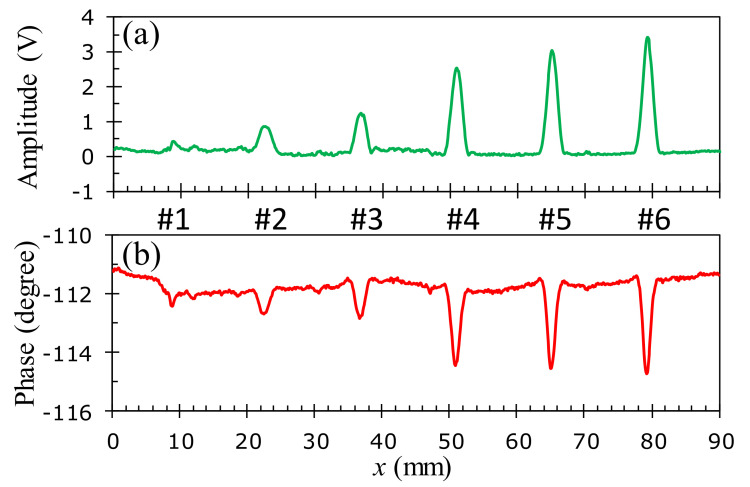
Inspection of cracks buried at *h* = 0.285 mm at the 30 kHz optimal excitation frequency on an aluminum specimen: (**a**) amplitude variation, (**b**) phase angle variation.

To assess the detection capability of the developed probe and investigate the effect of the size and position of flaws on the test sample, the experimental studies with the 1D-scan (B-scan) process are performed on the aluminum samples described in Figure 6. The specimens in Figure 6b–d of the buried depths of *h* = 0, 0.095, and 0.285 mm are inspected at the corresponding optimal excitation frequencies of 45, 40, and 30 kHz, respectively. Figure 16, Figure 17 and Figure 18 show the relationship between the amplitude and phase of the output signals for the artificial crack-like flaws #1 to #6 at the buried depths of *h* = 0, 0.095, and 0.285 mm, respectively. It is found that the signal morphology variation in the amplitude and phase correlates strongly to the crack depth of the artificial flaws. The location of the crack defect is indicated by the peak position of the obtained signals and the crack depth can be distinguished by the magnitude of the amplitude or phase. All of the surface cracks with depths of 0.1, 0.3, 0.5, 1.0, 1.5, and 1.8 mm can be clearly detected, as shown in Figure 16. These crack-like defects are still detectable when they are buried at the depths of 0.095 and 0.285 mm beneath the aluminum tapes, as shown in Figure 17 and Figure 18, respectively.

**Figure 19:**
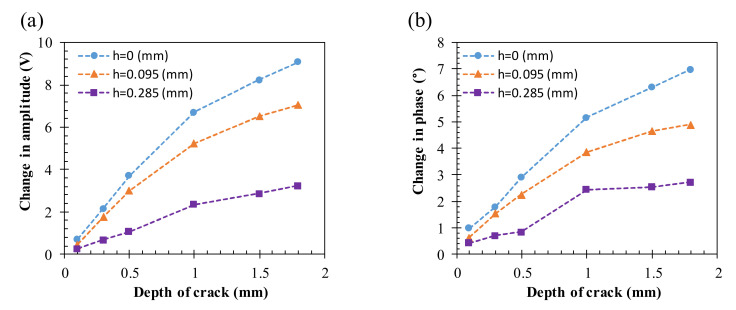
Relation of the amplitude and phase signals to crack depth for flaws buried at *h* = 0, 0.095, and 0.285 mm on an aluminum specimen. (**a**) The change in the amplitude signal and (**b**) the change in the phase signal.

To investigate the relationship between the EC signals and the crack depths, the peak values of the amplitude and phase in Figure 16, Figure 17 and Figure 18 are analyzed for various buried depths, as shown in Figure 19. It is found that both the changes in amplitude and phase increase linearly with the crack depth for the surface and buried cracks when the crack depth is less than 1 mm. For both the amplitude and phase signals, the detection of the surface cracks exhibits the highest sensitivity and the sensitivity decreases gradually with the increasing buried depth of 0.095 and 0.285 mm, as shown in Figure 19a,b. The experimental results are qualitatively in agreement with simulated signals based on the design parameters of the proposed probe.

The obtained signals are noisier for subsurface cracks with an increasing buried depth. For the deepest crack buried at *h* = 0.285 mm, the change in the amplitude and phase signals of the 0.1 mm deep crack is not obvious as it approaches the signal noise level. This means that the detection limit in the crack depth is around 0.1 mm at the buried depth of 0.285 mm. Increasing the excitation amplitude may help to improve the detection limit as long as the response of the GMR sensor is still within the linear range under the enhanced excitation. Table 3 shows the signal-to-noise ratios (SNRs) of the amplitude and phase signal for inspecting a 1.8 mm deep crack buried at different depths of 0, 0.095, and 0.285 mm. It is found that the SNR of the surface crack detected by the proposed probe is 37.8 dB for the amplitude signal and 50.9 dB for the phase signal. The SNR gradually reduces when increasing the buried depth, but the existence of the crack is still observable at a buried depth of up to 0.285 mm.

### 5.4. Determination of Crack Orientation

**Figure 20:**
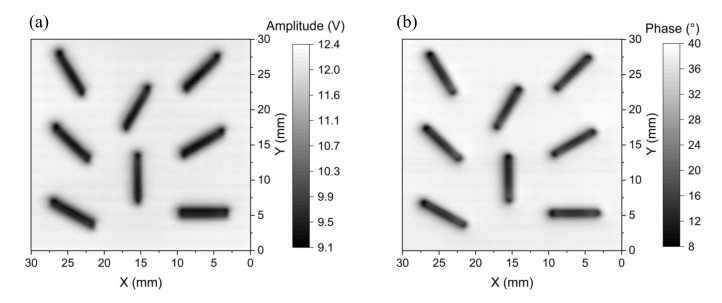
2D images of (**a**) amplitude and (**b**) phase at the 60 kHz excitation frequency for cracks of various orientations on a PCB.

For measuring crack orientation, several EC techniques have been developed to improve the angular accuracy, such as an excitation inducer with two orthogonal wires to generate a pseudo-rotating magnetic field [35] and a triple-coil eddy-current sensor operated on the thin-skin regime [36]. These methods can measure any crack orientation without the mechanical rotation of the probe or the test sample. Although crack orientation may be determined by the two-dimensional (2D) eddy-current images [20,27], the achievable precision of the crack orientation extracted from the eddy-current images depends mainly on the probe design, as well as on the configuration of the magnetic sensor. To test the performance for detecting crack orientation with our EC probe, the cracks with different angles of 0°, 30°, 45°, 60°, 90°, 120°, 135°, and 150° on the PCB sample in Figure 7 are used to obtain the 2D images, as shown in Figure 20, in which the images are taken at the excitation frequency of 60 kHz by moving the sample under the fixed EC probe to perform a surface scan (C-scan). The amplitude and phase images are shown, respectively, in Figure 20a,b. The orientation of cracks can be extracted from the amplitude and phase images, as shown in Table 4. It can be seen that the directions of the cracks on the 2D images approximate those of cracks on the tested sample with angle errors of no more than 1.6° and 2.6°, respectively, for the amplitude and phase images. The results indicate that the direction of cracks can be clearly determined with high accuracy and reliability. Besides, the shape and size of cracks can be determined directly from the phase images, of which the spatial resolution is about 1 mm. The result suggests that the proposed probe is useful for measuring the geometrical features of cracks of any orientation. The performance for determining crack orientation of our EC probe is better than the probes based on the AMR planar gradiometer with the baseline of 4 mm [27] and the absolute GMR probe [20], in which the reported experimental data reveal that the cracks aligned parallel to the baseline of the planar gradiometers or the sensing direction of the absolute probes are not detectable. Even if the test sample is rotated with different angles, the information is still insufficient for accurately determining the crack orientations that can be clearly observed by our probe without rotating the test sample or applying any algorithm and image-processing technique.

### 5.5. Spatial Resolution: Flaw Inspection on the Multilayer Printed Circuit Board

**Figure 21:**
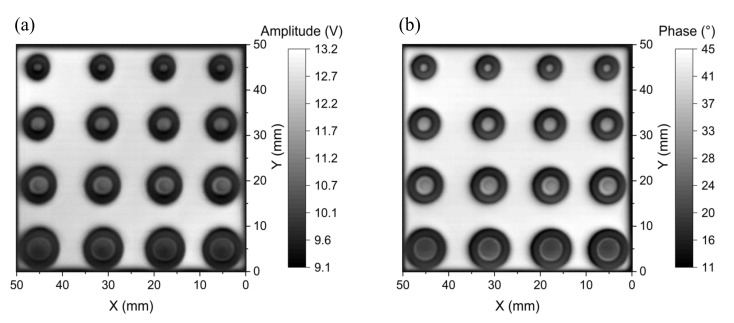
2D images of (**a**) amplitude and (**b**) phase at the 60 kHz excitation frequency for the surface flaws on the two-layer PCB.

**Figure 22:**
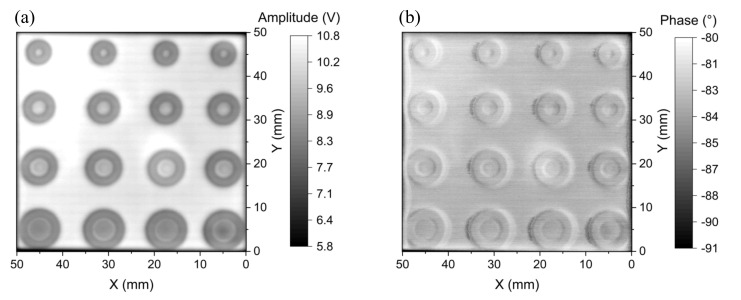
2D images of (**a**) amplitude and (**b**) phase at the 40 kHz excitation frequency for the flaws buried at the second layer of the three-layer PCB sample.

To evaluate the spatial resolution of the proposed probe, the 2D EC images of the multilayer PCB samples with artificial circular defects of the shape described in Figure 8 are analyzed. For detecting the surface flaws of the two-layer PCB sample, the amplitude and phase images are taken at 60 kHz, as shown in Figure 21. It can be seen that the shape and size of each of the machined flaws are clearly identified in both the amplitude and phase images. The geometric characteristics of the flaw, including an inside circular hole, a ring-shaped flaw, and the metallic ring between them are mostly discernible in Figure 21. The metal losses are indicated by the deep grey color, which corresponds to the lower amplitude or phase. Although the geometric features of all flaws are detected, the change in the signal intensity over the inside circles of the flaws F_1_ and F_2_ is not as clear as the flaws F_3_ and F_4_. Further reduction in the diameter of the excitation coil may improve the performance for detecting such small and complex-shaped defects. For detecting the surface flaws on the two-layer PCB, the proposed probe has achieved an SNR up to 30.1 dB in the amplitude image and 67.1 dB in the phase image. The performance of the developed probe for taking the 2D EC images of the circular flaws buried at the depth of 0.18 mm on the three-layer PCB is shown in Figure 22, where the excitation frequency is 40 kHz. It can be seen that the quality of the amplitude and phase images is noticeably blurred, while the flaw profile can still be observed in the amplitude image with a signal-to-noise ratio of 26.9 dB. The geometrical features of flaws F_3_ and F_4_ are clearly detected, but the inside circles of flaws F_1_ and F_2_ are not distinguished from the metallic ring around them. This means that the spatial resolution limit of the probe for detecting the metallic ring is not better than 1 mm when they are buried at the depth of 0.18 mm in the three-layer PCB.

**Figure 23:**
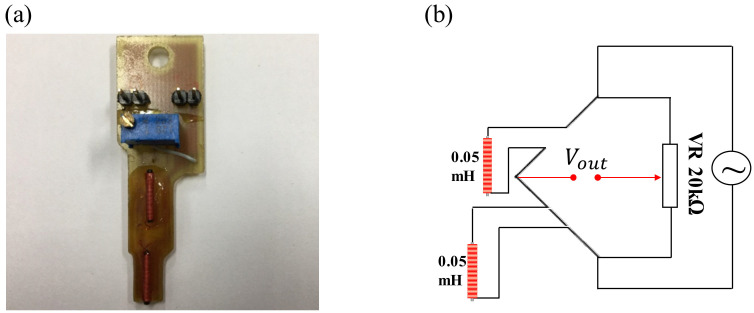
The coil-based probe: (**a**) the photograph and (**b**) circuit diagram of the coil probe.

**Figure 24:**
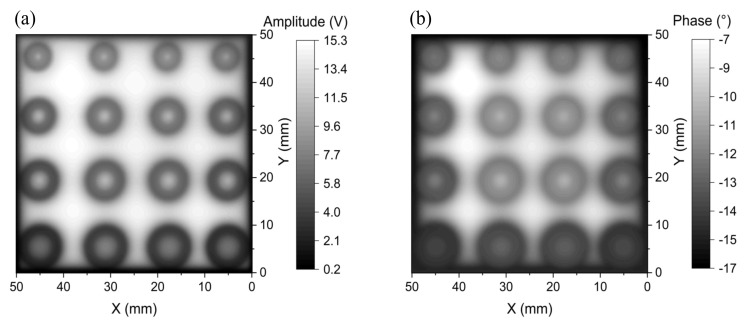
2D images of (**a**) amplitude and (**b**) phase at the 60 kHz excitation frequency of the coil-based probe for detecting the surface flaws on the two-layer PCB.

The performance of the proposed GMR EC probe is also compared with the coil-based half-bridge probe consisting of two ferrite-core coils with 1.5 mm diameters. In the coil probe, the two coils (a sensing coil and a reference coil) are in parallel with two resistors to form a full bridge, as shown in Figure 23. The two resistors are the two branches of a 20 kΩ variable resistor, which can balance the bridge by minimizing the background output of the bridge. With the coil probe, the obtained amplitude and phase images at 60 kHz for detecting the surface defects on the two-layer PCB are shown in Figure 24. Contrary to the images taken by the GMR EC probe in Figure 21, it is found that the features of the defect are better observed in the amplitude image in comparison with the phase image. Although the coil-based probe has high SNRs of 48.6 and 60.8 dB, respectively, for the amplitude and phase images, the metal ring between the inside circular hole and the ring-shaped defect in flaws F_1_, F_2_, F_3_, and F_4_ cannot be distinguished and the shapes of minor defects are not discernable in either the amplitude and phase images. From Figure 21 and Figure 24, it can be seen that the spatial resolution of the EC image of the surface flaws obtained by the coil-based probe at 60 kHz is poorer than the developed GMR EC probe with an even larger excitation coil. The surface flaws are not visible even with the coil-based probe operated at 45 kHz or lower, which means that the frequency above 45 kHz is already high, which results in a significant attenuation in the eddy-current signal from the subsurface flaws. As the sensitivity of the coil-based probe is insufficient below 60 kHz, the only way to improve the detectivity and spatial resolution is to further reduce the coil diameter and operate the coil-based probe at an even higher frequency, but this would make it more difficult to detect the subsurface flaw.

**Figure 25:**
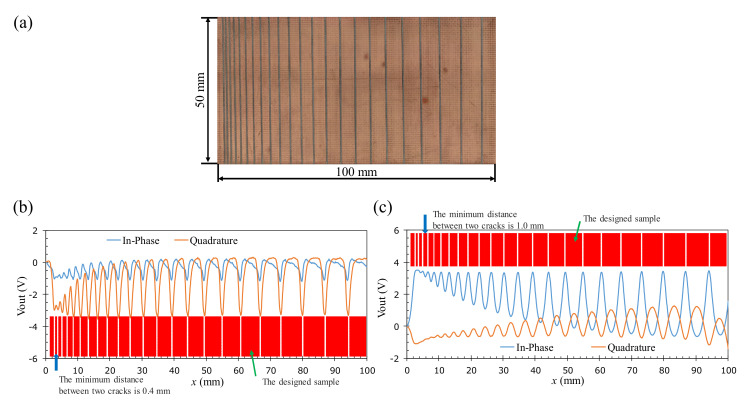
Determining the spatial resolution of the probe. (**a**) The photograph of the sample with the 23 artificial cracks with the increasing distance between cracks of a 0.3 mm arithmetic progression, (**b**) the 1D scanning result of the proposed probe, and (**c**) the 1D scanning result of the coil-based probe.

To compare the proposed probe and the coil-based probe in terms of spatial resolution, signal sensitivity, and the trade-off between spatial resolution and signal sensitivity, several other experiments were conducted. For determining the spatial resolution, a new sample of the two-layer PCB with the 23 artificial cracks is used, in which the distance between the cracks is gradually increased from 0.4 mm to 6.7 mm with an arithmetic progression of 0.3 mm. The experimental data are shown in Figure 25. In this way, the spatial resolution is determined by the minimum distance between the adjacent cracks that are observable by the EC probe. The maximum amplitude occurs for the cracks with the maximum spacing, which is used to calculate the signal-to-noise ratio to compare the sensitivity of the GMR and coil-based probes to the crack-like flaw. When boosting the spatial resolution by reducing the size of the sensor or excitation coil, the sensitivity of the probe will decrease because of the increased noise level. Therefore, the spatial resolution and sensitivity cannot be maximized at the same time. To quantify this situation, the S/E size ratios, which are defined as the ratio between the sizes of the sensor and excitation coil, are calculated and found to be 0.13 and 1, respectively, for the GMR and coil-based probes, as shown in Table 5. These ratios can be taken as the indicator of the trade-off between spatial resolution and signal sensitivity. According to the observed result, it can be seen that the proposed probe has achieved a high spatial resolution of 0.4 mm with a smaller S/E ratio. The high spatial resolution is achieved without losing too much SNR, which is indicated by the lower S/E ratio of the GMR probe.

## 6. Conclusions

We have proposed a novel design for a miniature half-bridge GMR gradiometer EC probe capable of locating the positions of flaws and estimating their geometrical features on a tested sample with high spatial resolution and sensitivity. It is able to detect small and complex-shaped defects on the surface and subsurface of highly conductive samples. The experimental results show that the proposed probe can detect the small crack with a 0.1 mm minimum depth buried at 0.285 mm deep beneath the surface of an aluminum sample. The proposed probe can determine the orientation of the cracks along any in-plane direction without rotating the test specimen or applying a reverse calculation algorithm and further image processes. From the 2D EC images taken by the axial gradiometer, one can identify the concentric circular flaw with a 0.75 mm feature size on the surface of a two-layer PCB sample. The achieved high spatial resolution and sensitivity of the proposed probe provides better performance in comparison with the coil-based probe, having a smaller diameter of 1.5 mm. The miniature GMR-based gradiometer is especially useful for detecting minor surface and subsurface defects, including mechanical cracks, corrosion, and short circuits, in the multilayer printed circuit boards.

## Figures and Tables

**Table 1:** Geometric parameters of the excitation coil.

Quantity	Dimensions
Inside dimensions	1.6 mm × 1.8 mm
Outside dimensions	2.9 mm × 3.1 mm
Height	1.42 mm
Diameter of wire	0.05 mm
Minimum lift-off *l*_0_	0.2 mm
Number of turns	252

**Table 2:** Geometrical Characteristic of Machined Holes on PCB.

Defects Number	*D*_1_ (mm)	*D*_2_ (mm)	*D*_3_ (mm)
F_1_	0.75	1.75	4.75
F_2_	1.5	3.5	6.5
F_3_	2.0	4.5	7.5
F_4_	3.5	5.5	8.5

**Table 3:** SNR in amplitude and phase for a 1.8 mm deep crack buried at different depths.

STT	Buried Depth (mm)	Frequency (kHz)	Amplitude SNR (dB)	Phase SNR (dB)
1	0	45	37.8	50.9
2	0.095	40	35.5	44.2
3	0.285	30	29.8	36.6

**Table 4:** Orientation of cracks extracted from 2D images.

Cracks Number	Real Angle (°)	Angle from Amplitude Image (°)	Angle from Phase Image (°)	Angle Error for Amplitude Image (°)	Angle Error for Phase Image (°)
A_1_	0	0.6	0.3	0.6	0.3
A_2_	30	30.4	29.8	0.4	0.2
A_3_	45	44.9	44.1	0.1	0.9
A_4_	60	59.8	58.5	0.2	1.5
A_5_	90	90.6	90.8	0.6	0.8
A_6_	120	121.6	122.6	1.6	2.6
A_7_	135	136	136.8	1.0	1.8
A_8_	150	150.8	151.1	0.8	1.1

**Table 5:** The comparison between the proposed probe and the coil-based probe.

Probe	Spatial Resolution (mm)	SNR (dB)	S/E Size Ratio
Proposed probe	0.4	29.7	0.13
Coil-based probe	1.0	39.5	1.0

## Data Availability

Not applicable.
